# Interactions of malnutrition and immune impairment, with specific reference to immunity against parasites

**DOI:** 10.1111/j.1365-3024.2006.00897.x

**Published:** 2006-11

**Authors:** S HUGHES, P KELLY

**Affiliations:** Tropical Gastroenterology and Nutrition Group, University of Zambia School of Medicine Lusaka, Zambia

## Abstract

1. Clinical malnutrition is a heterogenous group of disorders including macronutrient deficiencies leading to body cell mass depletion and micronutrient deficiencies, and these often coexist with infectious and inflammatory processes and environmental problems.
2. There is good evidence that specific micronutrients influence immunity, particularly zinc and vitamin A. Iron may have both beneficial and deleterious effects depending on circumstances.
3. There is surprisingly slender good evidence that immunity to parasites is dependent on macronutrient intake or body composition.

## INTRODUCTION

It is well recognized that the relationship between malnutrition and infection is an intimate one, and it is often assumed that this is because of impaired immune function. Management guidelines for treatment of malnutrition in children explicitly recognize that treatment of overt and occult infection is a first step in breaking the cycle of infection, malnutrition, and immune impairment. In this review, we shall explore one direction of this complex interaction by trying to answer the question ‘what is the effect of malnutrition on immunity?’ We will deal only with undernutrition, not with the immunological consequences of overnutrition. We must also point out that there are simply too few data to permit us to analyse the impact of each type of nutritional deficiency on the many pathways involved in immunity against parasites. Instead, we will try to draw broad conclusions from such information as does exist.

We can restate the above question by considering some recent observations on the pathogenicity of two protozoa. In the course of a randomized controlled trial of the effect of an elemental diet on the outcome of severe diarrhoea–malnutrition in Zambian children (1), we submitted faecal samples for parasitological analysis at the beginning and at the end of the trial. For 1 month, 200 children were treated with either routine nutritional rehabilitation or an elemental diet (i.e. a diet in which all the macronutrients are broken down to amino acids, oligosaccharides and simple lipids). At the beginning of the trial all these children had persistent diarrhoea, which was an entry criterion. At the end of the trial all 161 survivors were free of diarrhoea. But the prevalence of pathogenic protozoa was only modestly reduced at the end of the trial compared to the baseline coprological analysis. Initially the prevalences of *Cryptosporidium parvum* and *Giardia intestinalis* were 24% and 6%, respectively, but after treatment they were 13% and 8%, respectively (M. Mwiya and S. Sianongo, unpubl. obs.). In other words, children with persistent diarrhoea who had had pathogens at the beginning of the trial became convalescent carriers. This recovery from diarrhoea was very likely to have been due to a nutritional intervention even though the protozoa were still present. There is very good evidence to attest to the fact that these species are pathogenic and in this and other studies *C. parvum* has been shown to be an independent predictor of mortality. We are led to conclude that improving nutrition restored some aspect of host defence, and this somehow improved the barrier function of the intestinal mucosa against potential pathogens. Thus, the expression of virulence is to some extent determined by host defences, and this can be modulated by nutritional status.

So our question becomes three. First, what are the major immunological defects in malnutrition that might increase susceptibility to parasitic infection? Second, what is it in the immune response that improves on nutritional rehabilitation? Third, which nutrients are most important for any of these effects? We will begin with a sketch overview of immunity against parasites and what we mean by ‘malnutrition’, then consider these three questions in turn.

## OVERVIEW OF PARASITE IMMUNITY

While other articles in this edition will cover much of this subject in more detail, we will sketch out the salient features of immunity against parasites in order to provide a framework in which failure in malnutrition can be considered. Prevention of infection relies predominantly on barrier function and innate immunity, whereas clearance of an established infection requires either a successful humoral response (e.g. trypanosomiasis) or a successful cell-mediated immune response (e.g. schistosomiasis).

Parasite immunity builds up gradually, with the most severe complications generally apparent in the youngest and immunologically naïve. As immunity develops through repeated exposure, disease and infection become less common. For example, in malaria, immunity leads to protection from death by 5 years, but infection leading to asymptomatic parasitaemia occurs well into adult life (2). The mechanisms of immune-mediated resistance to disease and to infection remain an area of great interest but of limited understanding. The initial encounter between host and parasite usually involves inoculation or penetration into the bloodstream, or contact with a mucosal surface. In the first of these, the blood- or lymph-dwelling forms are exposed to soluble molecules and phagocytic cells. In the second, penetration into mucosal cells, for example in the intestine, immediately exposes the penetrating stage of the parasite to epithelial cells and to dendritic cells (DCs) in the Peyer's patch.

The initiation of immune responses requires recognition of ligands on the parasite by receptors of the innate immune system or of the adaptive immune system. The sensory arm of innate defence pathways includes receptors on cells such as macrophages, neutrophils, and natural killer (NK) cells, including macrophage mannose receptor, scavenger receptors and Toll-like receptors (TLRs), and soluble receptor molecules (mannose-binding lectin, MBL, and complement). MBL deficiency has been associated with cryptosporidiosis (3) and probably with malaria (4). The sensory arm of the adaptive immune system includes the B-cell receptor (immunoglobulin) and the T-cell receptor for which the ligand is antigen presented by HLA molecules on antigen-presenting cells. Ligands of innate immune receptors (i.e. T-cell-independent immune responses) are molecules characterized by repeated motifs that are recognized in a class-specific manner, so-called pathogen-associated molecular patterns (PAMPs). Thus, innate immune receptors recognize prokaryotic and viral molecules that exhibit these repetitive patterns. The receptors that recognize these motifs are referred to as pattern recognition receptors and they are ‘hard-wired’ into the genome.

Ligands of adaptive immune receptors do not display such molecular conformity and can distinguish more subtle nonself molecular characteristics such as specific protein sequences. Dendritic cells possess multiple innate receptors and interact with multiple cell types before committing specific T cells to activation. DCs are thus at the interface of innate and adaptive immunity and their signalling to T cells determines the type of immune response that will be generated. Their capacity to receive signals from both systems largely explains how adjuvants (which are ligands for innate receptors) augment adaptive immunity. There is emerging evidence that parasite components can interact with TLRs and thus with innate immunity. *Schistosoma* egg antigen regulates DC activation in response to TLR activation (5), *Plasmodium* haemozoin activates TLR9 (6), and *C. parvum* interacts with TLRs 2 and 4 (7).

Innate effector mechanisms include phagocytosis, NK cell killing, complement-mediated lysis and opsonization, and antimicrobial peptides. Much more remains to be learned about all these elements in defence against parasites as they have received much less attention from immunologists than adaptive immune responses. What is beyond doubt is that the capacity to overcome or subvert innate host defences (for example, complement by parasites (8)) is an important element of pathogenicity (9). At mucosal surfaces, this can be recognized as crossing the epithelial barrier. With certain exceptions, such as toxin-secreting organisms, a pathogen could be defined as an organism that has escaped the compartmentalization of host and commensal through by-passing innate defences.

Adaptive immune elements include antibody responses, particularly IgG that has a modest role in clearance and protection against *Plasmodium, Toxoplasma, Trypanosoma*, and CD4 T-cell responses that are the dominant responses in clearance and protection against the above and against *Leishmania* and *Schistosoma* spp. IgE-dependent mast cell and eosinophil responses play a major role in expulsion of helminths. CD4 T cells in small intestinal epithelium have been shown, at least in adoptive transfer experiments in mice, to play a major role in clearance of *C. parvum* (10). Both innate and adaptive systems can lead to inflammation, but inflammation is not the dominant response to metazoan parasites, which relies on specific pathways of clearance.

## MEANING OF ‘MALNUTRITION’

The term ‘malnutrition’ describes any disorder resulting from an inadequate or unbalanced diet, or a failure to absorb or assimilate dietary elements. It is a broad term and can even refer to overnutrition. But in terms of the health of populations in tropical countries and their susceptibility to infectious disease, we are interested in the effects of inadequate intake or absorption/assimilation of macronutrients and micronutrients. Macronutrients, present in the diet in gram or kilogram quantities, are the constituents of body tissues, carbohydrate, proteins, fats and nucleic acids (though deficiency of nucleic acids has not been described). Micronutrients are present in much smaller quantities (milligram or microgram) and are required for specific metabolic functions. Examples of micronutrients are vitamins, and minerals such as calcium, iron, zinc, copper, selenium, and iodine.

Assessment of nutritional status is a complex subject beyond the scope of this article, but it can be divided into three elements: assessment of diet, assessment of body composition, and assessment of micronutrient status. As malnutrition has such a profound effect on functional performance, many nutritionists would also add that assessment of function (such as muscle strength, cognitive ability, quality of life) should be included.

Separate syndromes of severe malnutrition are recognized: severe wasting (marasmus), oedematous malnutrition (kwashiorkor), and the coexistence of oedema with severe wasting (marasmic kwashiorkor). Severe malnutrition is a result of two dominant processes: primary malnutrition (food deprivation) usually as a result of conflict or famine, and secondary malnutrition resulting from infectious, inflammatory, or malignant disease leading to anorexia and/or increased nutrient demand. In both of these situations, peripheral oedema may supervene but its pathogenesis is not understood and it is not clear if the presence of oedema has any implications for host defence. As medical/scientific research is carried out in peacetime in fairly stable settings, most of the work on host defence in malnutrition in humans has failed to dissect out the consequences of macronutrient and/or micronutrient depletion and the infectious and inflammatory processes that gave rise to it. This is a serious problem in the literature.

## WHAT ARE THE MAJOR IMMUNOLOGICAL DEFECTS IN MALNUTRITION?

There is a very large body of literature that attempts to define immunological dysfunction in malnourished patients, which we will deal with here, though probably the best evidence comes from intervention studies (see below). Studies reporting the findings in cohorts of children with severe malnutrition are difficult to integrate, as the studied groups are often incompletely described and when described well, clearly far from homogenous. There are difficulties in the definition of malnutrition, the identification of cause, and the comprehensive description of concurrent infections, which are often hidden yet are critical confounders. With more complex testing procedures, problems also exist with the definition of normal ranges for age-matched and infection-matched controls. Future studies will need to describe very carefully the groups studied with particular attention to infectious diseases, and have control data clearly identified to reduce bias and aid interpretation.

Our work in Lusaka addresses children and adults with malnutrition, HIV, and a broad spectrum of infectious diseases and gastrointestinal pathologies. In one recent study, we were not able to confidently identify a single case of primary malnutrition in a cohort of 84 severely malnourished children, as all had presented with history of either lengthy diarrhoeal disease, or pulmonary disease or were found to be HIV infected.

The most compelling evidence that malnutrition is associated with immunodeficiency comes from the descriptions of the infections in severe malnutrition. However, it must be remembered that infections are as much a cause of malnutrition as a consequence, and errors can be made ascribing cause and effect. Infection itself is known to have a negative effect on immunocompetence. Regarding infections in the severely malnourished, two additional facts must be considered. First, infection plays a very major role in the clinical presentation of severe malnutrition. Second, infection is often silent, as the febrile response to infection is often inadequate.

Many authors have aimed to study primary malnutrition, yet this is difficult. As an alternative, the most ‘pure’ form of human undernutrition amenable to study is anorexia nervosa. Although susceptibility to infectious disease is less in anorexia nervosa than in other forms of undernutrition (11), IL-2 synthesis was reduced by 49% in one study (12). However, T-lymphocyte populations were normal and lymphocyte proliferation in response to phytohaemagglutinin and concanavalin A was if anything increased (11). High circulating levels of IL-1β and TNF-α were observed in another study, together with reduced T-cell activation as expressed by CD2 and CD69 (13). These findings and others listed below leave us with considerable uncertainty as to whether it is the malnutrition *per se* that leads to the immune defects we describe, and in later sections we ask whether nutritional treatment can restore immune function.

Before examining the impact of malnutrition on elements of the immune system, it is important to first recognize that susceptibility to infection and associated mortality depends on other host factors also. Various aspects of barrier function become deranged in malnutrition, for example gastric acid secretion is reduced, leading to increased susceptibility to intestinal infection (14).

There is agreement that malnutrition worsens prognosis in AIDS patients (15), and in Lusaka we have confirmed that low body mass index is an independent predictor of death in the short term in patients with AIDS-related diarrhoea (16). Macronutrient support can improve survival in severely malnourished AIDS patients (17). However, it is not clear if this is an effect on immunological function.

Substantiation of malnutrition-related immunodeficiency is assembled from three distinct evidence bases.

1. Increased incidence or severity of infections. It should be noted that without evidence of increased susceptibility to, or severity of, infectious disease, abnormalities in laboratory assessments do not constitute an immunodeficiency.
2. Markers of immunodeficiency (laboratory or clinical, some of these are well validated, others much less so).
3. *In vitro* functional analysis of immune processes, i.e. dynamic assays.

Selected evidence, from studies of malnourished human subjects, for and against a malnutrition-associated immunodeficiency is presented in Table 1. Where possible, data have been collected from longitudinal nutrition intervention studies. Where this has not been possible, observational studies have been cited. The data represent well the breadth and depth of published findings, though the list of citations is not comprehensive.

**Table 1:** Evidence for elements of immunodeficiency in cases of malnutrition

Issue	Reported finding	Population studied	References
Infections			
* Pneumocystis jirovecii pneumonia*	High prevalence	SM – autopsy studies	18,19
* Mycobacterium tuberculosis* infection	High prevalence	SM – autopsy study	18
	‘Dissemination of disease’ including miliary and CNS tuberculosis	SM	20
Herpes viruses	High prevalence	SM – autopsy study	18
Acute respiratory tract infection	High prevalence	MM	21
Diarrhoea	High prevalence	MM	22
Malaria	Morbidity and mortality attributable to undernutrition	Malaria-infected cohorts	23
	Reduced risk of cerebral malaria		24
Leishmania	Malnutrition predisposes to visceral leishmaniasis	MM	25
Infection frequency	No increase	AN	11
Markers of immunodeficiency			
Thymolymphatic atrophy	Reduced bulk on autopsy	SM – autopsy studies	18,26
	Reduced bulk in life	SM (infected)	27
Tonsil size	Smaller size	MM, SM (infected)	26,28
Low leucocyte count	Present	Kwashiorkor	29
	Absent	AN	11
Low lymphocyte count	Present	AN, SM, MM	13,28,30
	Absent	AN, SM	11,31
Low T-cell count	Present	SM	27,30,32
	Absent	SM, AN	11,33,34
Low CD4 cell count	Absent	AN, SM	11,13,27,34
	Present	Kwashiorkor, ‘malnourished’	27,32
Antibodies	Raised levels	SM	33,35
	Lowered levels	AN	13
Specific antibody defects	Normal levels	MM	36
	Disparate results from different studies	Varying groups	37
Secretory IgA	Reduced levels	SM	38
	Raised production	Kwashiorkor	39
Acute phase protein response	Impaired responses	SM	40
	Unimpaired responses	SM	41,42
Complement	Reduced levels of haemolytic complement, reduced C3 and C4, reduced C3	SM, kwashiorkor, mild and moderate malnutrition	28,31,33,43
	Normal levels	AN	11
Functional testing			
Neutrophil function	Impaired phagocytosis	SM	44,45
	Normal or improved	MM and SM	46
	Opsonization and phagocytosis		
	Impaired microbicidal activity	MM and SM	45,46,47
	Impaired chemotaxis	SM	45
Monocyte function	Reduced cytokine (IL1, TNFα) production	SM	31,48,49
	Reduced phagocytosis of candida	SM	31
	Reduced lysis of candida	SM	31
T-cell function	Diminished Mantoux recall responses	SM	50
	Impaired BCG sensitization	Kwashiorkor	51
	Diminished multitest skin	MM	21
	Cell-mediated immunity (CMI) test		
	Partially impaired CMI	AN	11
	Reduced dinitrochlorobenzene sensitization	SM (infected), SM	26,30
	Reduced phyto(haem)agglutinin (PHA) mitogenesis	SM, AN, MM	13,26,28,45,52
	Increased PHA mitogenesis	AN	11
B-cell function (vaccine challenge and specific antibody responses)	Normal levels	MM	36

AN, anorexia nervosa; MM, moderate malnutrition; SM, severe malnutrition.

It is clear that there is much circumstantial evidence in support of malnutrition-associated immunodeficiency, but some evidence against it, and much uncertainty regarding cause and effect. Given the maelstrom of immune defects, it is tempting to consider these many elements of immune dysfunction as evidence of dysregulation rather than immunodeficiency; however, the disturbed processes remain to be uncovered. In addition, against classical immune dysregulation, there is no evidence of allergic disease in the severely malnourished and rarely is there a suggestion of autoimmune pathology.

**Figure 1:**
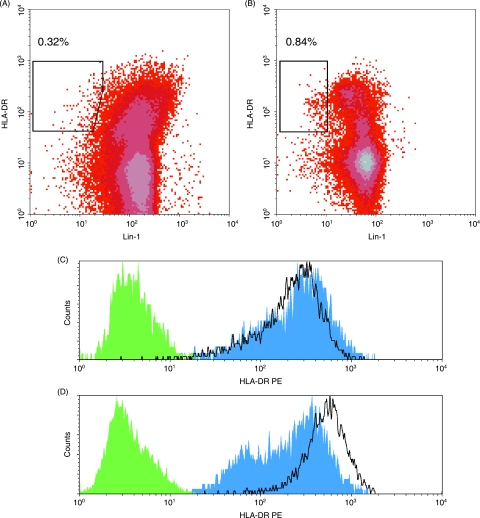
Evidence of depletion of dendritic cell (DC) number s and dysfunction of DCs in the child whose case is described in the text. A and B FACS (fluorescence activated cell sorter) plot of peripheral blood mononuclear cells (PBMCs) after initial selection by side scatter and CD45 expression. Each point represents one PBMC, and the intensity of staining with lineage markers (CD3, CD14, CD16, CD19, CD56) is shown on the *x* axis. DCs have little or no staining for these markers. HLA-DR staining is shown on the *y* axis; DCs have high DR staining and the box therefore includes those cells tha are likely DCs. A FACS plot on admission; B just prior to discharge, after good nutritional recovery and DC numbers have increased from 0·32% to 0·84% of PBMCs. C and D histograms of cultured DCs at rest (blue shading) and after stimulation with lipopolysaccharide (open histogram delineated by black line) which is expected to stimulate DCs; isotype control is shown as green histogram. C cells from admission sample fail to upregulate HLA-DR, but D after nutritional recovery the capacity to upregulate HLA-DR is restored.

Early data from our work in Lusaka suggest that DC function, which has not previously been addressed, may also be important, and may underlie some of the dysregulation described above. One child, a girl aged 20 months presented with a 3-month history of diarrhoea and a 5-day history of sores in the mouth, fever and cough. She was emaciated, and had pedal oedema. Her weight-for-height *z* score on admission was −3·61. She made a rapid recovery from her malnutrition and her diarrhoea ceased during her admission. Laboratory examination of her DCs on admission and then on recovery (Figure 1) identified a low DC count initially, which had risen at the time of her discharge. In concert with this finding was the discovery that she had an unusual phenotype to her cultured DC population. On stimulation with lipopolysaccharide (LPS), in contrast to normal phenotype, her DCs downregulated HLA-DR and CD86. Downregulation of HLA-DR expression reduces DC capacity to support a protective T-cell response to threat, thereby disabling the immune response.

## WHICH ELEMENTS OF THE IMMUNE RESPONSE RESPOND TO GLOBAL NUTRITIONAL REHABILITATION?

Standard nutritional rehabilitation for severe malnutrition now begins with blind antibiotic therapy, though in the past this was not routine. We have selected studies in which primary malnutrition was treated with nutritional therapy alone, though in many studies we cannot be certain that antibiotics were not given. Studies confirm that the initial finding of thymolymphatic atrophy resolves with renutrition (53), and in parallel, T-lymphocyte function as examined by cell proliferation and the tuberculin test improves (28,45,52). Note however, that repeated tuberculin tests will improve responses through the process of ‘vaccination’ alone. In addition, described defects of the innate immune system, such as complement levels (43), neutrophil microbicidal activity (45), secretory capacity for IgA transport into secretions (54), and monocyte production of cytokines (IL1β, TNFα, and IL6) (40,48), all improve on renutrition.

## WHICH NUTRIENTS COULD BE RESPONSIBLE FOR ANY IMPROVEMENT?

### Macronutrients

Clinical trials of nutritional rehabilitation and immune function are few. Although there are many trials of nutrition interventions and their effect on infectious disease, trials that show a successful improvement in nutritional status and a subsequent effect on measures of immunological function are very few. In one of the studies in anorexia nervosa referred to above, nutritional rehabilitation returned the increased mitogen responsiveness towards normal (11). Cytokine perturbations also returned to normal after re-feeding (13), but in both of these instances it is not possible to dissect out the influence of macro- and micronutrients.

In a trial in which Kenyan school children were randomized to several different food supplementation foods (meat-based, milk-based, vegetable oil-based or none), antibody titres to *Helicobacter pylori*, rotavirus, tetanus toxoid and malaria merozoite surface proteins showed very little change (55).

The effect of nutritional therapy on malaria has been unclear ever since the Murray team found in the 1970s that undernutrition protected against morbidity and mortality (24). This unexpected finding was borne out by studies in protein-deprived animals. Subsequent work has not really supported this contention, and a recent WHO analysis has, characteristically, attempted to quantify the proportion of malaria attributable to malnutrition (23). This more comfortable reading suggests that micronutrient deficiency plays a more significant role in immunity to malaria than macronutrient deficiency. However, we believe that the earlier work cannot be ignored, especially as it was carried out in a famine situation, which is a more ‘pure’ form of primary malnutrition.

It is well established that survival in AIDS is determined to a considerable degree by nutritional status (both macronutrient and micronutrient), and if this is through an effect on immune function one would expect to see improvements in CD4 count if weight gain can be achieved. Despite a careful search of several databases, no evidence for an effect of treatment using macronutrients on immune function in AIDS could be found (see also Macallan (15)). For example, parenteral nutrition improved nutritional status (body composition) compared to controls, but no assessment was made of immune function (17). There is no evidence that lipid supplementation is of benefit (56,57).

If there is a relationship between body composition and immune function, it might be mediated by leptin (58). Leptin is a 16 kDa protein hormone that was discovered as the missing gene product in the *ob/ob* obese mouse. Leptin, produced by adipose tissue, acts as a satiety signal: high levels are associated with body fat and levels decrease as fat tissue is lost during starvation. The leptin receptor has structural similarities to the IL-6 family of cytokines and leptin signalling is inhibited by SOCS-3 that regulates other cytokines. Macrophages from leptin-deficient mice are constitutionally activated and over-react in response to LPS, but their killing of *Escherichia coli* is impaired. Leptin-deficient mice also have lymphopenia and impaired delayed-type hypersensitivity (DTH). It is thus tempting to speculate that low circulating leptin in humans with wasting would lead to such T-cell and macrophage defects seen in both *ob/ob* mouse and in starved mice. There is evidence in *ob/ob* and starving mice that the immune dysfunction is mediated by leptin as leptin reverses the dysfunction (59). However, this has not been shown in humans, and the link between macronutrient depletion and the immune dysfunction remains tentative.

### Vitamin A

It has been clear that vitamin A has important anti-infective properties since 1932 when it was shown that it reduced case fatality from measles. Large studies in Ghana, Indonesia and elsewhere have confirmed that vitamin A has important effects in reducing adverse outcome from infectious disease in underdeveloped countries, particularly diarrhoea and measles (60). There are also two relevant clinical trials of the effect of vitamin A supplementation on malaria. The first, in Ghana, found no benefit on malaria morbidity (61), but the second, in Papua New Guinea (62), showed reduced malaria morbidity in children supplemented with vitamin A compared to placebo (relative hazard 0·70, 95%CI 0·57–0·87). Vitamin A supplementation may reduce placental infection (63). However, the outstanding question is: is this an effect on immune function or on some other aspect of host defence such as epithelial integrity?

In laboratory animals, vitamin A polarizes the immune response towards Th2 (64,65), acting through retinoic acid, its principal oxidative metabolite. Retinoic acid also boosts the antitetanus antibody response (66). However, evidence of an immune booster effect in humans is much less clear. This evidence has recently been thoroughly reviewed (67). To summarize this evidence (67), there is evidence that intestinal epithelial integrity is improved by vitamin A (68), but not of improved antimicrobial properties in breast milk, and no evidence of improved barrier function in the vagina. There is very preliminary evidence of reduced secretion of TNF-α and IL-6 when challenged by specific pathogens. There is some evidence of a beneficial effect in raising CD4 counts in HIV-infected children but not in adults. Neither is there conclusive evidence of effects on cytokine production or lymphocyte function, but antibody responses to tetanus toxoid may be enhanced if the vitamin A is given before the vaccine (67). When contrasted with the highly significant effects of vitamin A in reducing childhood morbidity and mortality, particularly from measles and diarrhoea, the very uncertain evidence of effects on immune competence is striking. It seems likely on the basis of current evidence that epithelial or barrier integrity is an important part of the effect of vitamin A. Furthermore, addition of a vitamin A supplement to a supplement of vitamins B, C, and E given to HIV-infected pregnant women detracted from the benefit attributable to the supplement (69) so the effects of vitamin A, even if mediated by augmented cell-mediated immunity, are complex and can be disadvantageous.

### Zinc

There is abundant clinical evidence that zinc is a critically important nutrient for the proper functioning of the immune system. Zinc is effective in prevention of diarrhoea: a recent review [6] of nine trials showed significant reductions in diarrhoea incidence, and all showed a reduction of some magnitude (70). Similar benefits were also found for pneumonia and malaria, though fewer trials are available for analysis. Zinc also gives a 42% (95%CI 10–63%) reduction in treatment failure or death from diarrhoea (71), though high doses may be detrimental (72). A meta-analysis of clinical trials of zinc supplementation in prevention of malaria concluded that a reduction in incidence of 36% (95%CI 9–55%) might be possible (23), but in one trial, there was no benefit on malaria incidence or severity at all (73). Thus, it appears that zinc supplementation is clinically effective in reducing morbidity and mortality due to diarrhoeal disease and malaria in children. But is this an effect on immunity or host defence or something else?

There are two lines of evidence that suggest that zinc deficiency adversely affects immune function and that supplementation improves it. First, in humans there are data from the 1970s which, though not conclusive, support this contention. Children with acrodermatitis enteropathica, a congenital defect of zinc absorption, have thymic atrophy, lymphopenia, reduced lymphocyte response to mitogens, reduced DTH, and reduced immunoglobulin responses (74). Many other reports of immune defects in zinc-deficient patients are difficult to interpret because of comorbid processes (e.g. renal failure) which could themselves impair immunity. But in an important study in Indian children with diarrhoea, zinc supplementation increased numbers of circulating CD3 and CD4 cells, but not CD8 cells, B cells or NK cells (75). In terms of innate immunity, Paneth cells, which synthesize antimicrobial molecules for innate defence of the small intestine in humans, are also dependent on zinc (76,77). Second, zinc deprivation of mice for as little as 30 days reduced cell-mediated immunity, DTH, antitumour immunity, and antibody responses by up to 80% (78). Challenging zinc-deficient animals with low doses of *Trypanosoma cruzi* or intestinal nematodes resulted in death (79). The deficiency state was associated with reduced numbers of lymphocytes due to impaired lymphopoiesis, but the production of antibody by each cell was not impaired. Furthermore, while zinc deficiency had marked effects on lymphoid cells, there was no effect on myeloid cells, and this lead Fraker *et al*. (78) to advance a fascinating and potentially very important theory. This is that maintenance of lymphocyte populations is very expensive in terms of zinc and other nutrients, and that in the face of nutritional stress innate defence is maintained at the expense of adaptive immune responses. The Fraker theory is very attractive and deserves much further work. If true, the ramifications for management of infectious disease in malnourished patients could be considerable.

However, there is evidence that NK cell function and phagocytosis by macrophages are also impaired in zinc deficiency, and this may be a consequence of reduced oxidative burst capacity, for example in trypanosomiasis (80). Zinc supplementation of mice during *Plasmodium berghei* infection reduced markers of oxidative stress (81), but the significance of this is not clear. Zinc itself induces release of IL-1, IL-6, TNF-α, and IFN-γ in macrophages but not T cells, and high supraphysiological concentrations suppress T-cell functions (82). Early data suggest that zinc is important for maintenance of antimicrobial peptide delivery in the small intestine (77,83).

The most definitive evidence that zinc deficiency is critical for immune function in humans comes from experimental zinc deficiency induced by dietary restriction in human volunteers (84). Deficiency reduced thymulin levels in blood, and reduced the CD4/CD8 ratio. Zinc deficiency also reduced synthesis of Th1 cytokines IL-2 and IFN-γ, but not the Th2 cytokines IL-4, IL-6, and IL10. NK cell activity was also reduced in the volunteers on a zinc-deficient diet.

### Iron

In studies in iron-deficient humans, iron deficiency has been associated with defects in both adaptive and innate immunity, and these are reversible with iron therapy (85). Adaptive immune defects include reduced T-cell numbers, reduced T-cell proliferation, reduced IL-2 production by T cells, reduced MIF production by macrophages, and reduced tuberculin skin reactivity. Innate immune defects include reduced neutrophil killing, probably due to reduced myeloperoxidase activity and impaired NK cell activity.

However, the picture is far from simple. Lactoferrin in human milk chelates iron and inhibits bacterial proliferation by depriving the bacteria of an essential nutrient. The bacteriostatic effect of human milk is abolished by iron therapy (86), so that iron therapy would be expected to increase neonatal intestinal infectious disease. In milk-drinking nomads, iron therapy was associated with an increase in *Entamoeba histolytica* infection, possibly due to saturation of the milk transferrin that overcomes the protective effect (87).

The same group had previously noted recrudescence of malaria and schistosomiasis in nomads treated with iron (88). An overview of iron supplementation studies in malarious regions included 11 trials (85). Five of nine trials in which clinical malaria was assessed showed a deleterious effect, and no trials showed benefit. Respiratory infections and other infectious morbidity were also, if anything, increased (though diarrhoeal disease was not). A recent observational study in Kenya (89) indicated that the incidence of clinical malaria was lower among iron-deficient children (IRR 0·70, 95%CI 0·51–0·99). This deleterious effect of iron supplementation on infectious disease has not been observed in clinical trials in nonmalarious regions (85), though there is evidence that dialysis and multiply transfused patients with iron overload have immune defects (90). Finally, and fairly conclusively, a recent large study seems to confirm previous observations that iron supplementation worsens infectious disease morbidity and mortality by 11–15% (91) and, taken together, the evidence is that this effect is real and important.

In summary, it is difficult to draw a firm conclusion as to whether iron status contributes to the impaired immunity to parasites seen in malnutrition. There is evidence of T-cell and innate immune impairment in iron deficiency, but supplementation (i.e. supraphysiological intakes of iron) seems to worsen susceptibility to malaria and possibly to other infectious diseases.

### Other antioxidant molecules

Selenium is an important antioxidant that has been shown to have wide-ranging immunostimulant effects in macrophages and T and B cells in humans (92). However the evidence for this rests on a very small number of primary publications (93,94). The most compelling recent evidence is an example of the sort of functional immunological testing that is all too rare in this field (95). Twenty-two British volunteers with low plasma selenium concentrations were given modest doses of a selenium supplement (up to 100 µg/day) or placebo, then were challenged with oral polio vaccine and immune responses to the vaccine determined (95). Selenium supplemented volunteers showed increased T-cell proliferation, and higher interferon-γ and IL-10 production by T cells 7 days after vaccination. They also showed more rapid clearance of the virus from stool. The situation is very similar for vitamin E, in which there is much interest, but for which the evidence base is narrow. In one clear-cut study in elderly people, vitamin E supplementation for 4 months increased DTH responses and increased antibody titres to clinically relevant vaccines (hepatitis B, tetanus) but not immunoglobulin levels or T or B cell numbers (96). There are no data to our knowledge of the effect of these micronutrients specifically on immune responses to parasites, but these data suggest that antioxidant nutrients are likely to be important in maintaining immunity.

## CONCLUSIONS

There is evidence that malnutrition impairs elements of adaptive and innate immunity which would be important for defence against parasitic infections, although evidence of increased incidence or severity of parasitic infections in malnourished humans is fairly limited. The evidence that this immune dysfunction is attributable to deficiency of protein or other macronutrients is weak; we find it unconvincing and conclude that it has been overstated in the past on the basis of poorly controlled studies. On the other hand, there is good evidence of links between micronutrient deficiencies and immune impairment. This evidence is strongest for zinc, deficiency of which leads to impairment of both innate and T-cell responses. The evidence that antibody responses are impaired in any malnourished state is much less convincing. Given the very heavy burden of infectious disease around the world, and its massive contribution to illness and premature death, this field warrants much greater attention. As primary malnutrition is usually associated with famine, conflicts and population displacement, and confounding factors in secondary malnutrition are inevitable, observational studies are difficult to interpret. Study of patients with anorexia nervosa could still give much useful information on the impact of macronutrient depletion. However, the most useful information will be derived from specific controlled interventions in volunteers and in patients.
